# Inconsequential role for chemerin‐like receptor 1 in the manifestation of ozone‐induced lung pathophysiology in male mice

**DOI:** 10.14814/phy2.16008

**Published:** 2024-04-17

**Authors:** Richard A. Johnston, Albert W. Pilkington, Constance L. Atkins, Theresa E. Boots, Philip L. Brown, William T. Jackson, Chantal Y. Spencer, Saad R. Siddiqui, Ikram U. Haque

**Affiliations:** ^1^ Health Effects Laboratory Division, National Institute for Occupational Safety and Health, Centers for Disease Control and Prevention United States Department of Health and Human Services Morgantown West Virginia USA; ^2^ Section of Pulmonary, Critical Care, and Sleep Medicine, Department of Medicine, School of Medicine West Virginia University Morgantown West Virginia USA; ^3^ Division of Critical Care Medicine, Department of Pediatrics McGovern Medical School at the University of Texas Health Science Center at Houston Houston Texas USA; ^4^ Department of Integrative Biology and Pharmacology McGovern Medical School at the University of Texas Health Science Center at Houston Houston Texas USA; ^5^ Division of Pulmonary Medicine, Department of Pediatrics McGovern Medical School at the University of Texas Health Science Center at Houston Houston Texas USA; ^6^ Section of Pediatric Pulmonology, Department of Pediatrics Baylor College of Medicine Houston Texas USA; ^7^ Division of Critical Care, Department of Pediatrics Sidra Medicine Doha Qatar

**Keywords:** airway hyperresponsiveness, chemerin‐like receptor 1, elastic recoil, lung, ozone

## Abstract

We executed this study to determine if chemerin‐like receptor 1 (CMKLR1), a *G*_i/o_ protein‐coupled receptor expressed by leukocytes and non‐leukocytes, contributes to the development of phenotypic features of non‐atopic asthma, including airway hyperresponsiveness (AHR) to acetyl‐β‐methylcholine chloride, lung hyperpermeability, airway epithelial cell desquamation, and lung inflammation. Accordingly, we quantified sequelae of non‐atopic asthma in wild‐type mice and mice incapable of expressing CMKLR1 (CMKLR1‐deficient mice) following cessation of acute inhalation exposure to either filtered room air (air) or ozone (O_3_), a criteria pollutant and non‐atopic asthma stimulus. Following exposure to air, lung elastic recoil and airway responsiveness were greater while the quantity of adiponectin, a multi‐functional adipocytokine, in bronchoalveolar lavage (BAL) fluid was lower in CMKLR1‐deficient as compared to wild‐type mice. Regardless of genotype, exposure to O_3_ caused AHR, lung hyperpermeability, airway epithelial cell desquamation, and lung inflammation. Nevertheless, except for minimal genotype‐related effects on lung hyperpermeability and BAL adiponectin, we observed no other genotype‐related differences following O_3_ exposure. In summary, we demonstrate that CMKLR1 limits the severity of innate airway responsiveness and lung elastic recoil but has a nominal effect on lung pathophysiology induced by acute exposure to O_3_.

## 1 INTRODUCTION

Human subjects who inhale ozone (O_3_), an inorganic, highly reactive oxidant gas and criteria pollutant, exhibit airway hyperresponsiveness (AHR) to acetyl‐β‐methylcholine chloride (methacholine), cough, dyspnea, inspiratory chest pain, decrements in lung function [forced expiratory volume in 1 s and forced vital capacity], decreases in tidal volume, and increases in specific airway resistance and breathing frequency (Balmes et al., 1996; Foster et al., 2000; Hazucha et al., 1989). O_3_ also causes (1) lung injury, which is characterized, in part, by lung hyperpermeability and by desquamation of ciliated airway epithelial cells and (2) lung inflammation, which manifests as increased numbers of neutrophils and eosinophils in bronchoalveolar lavage (BAL) fluid and altered expression of genes involved in numerous biological processes, including cell adhesion, cellular defense, chemotaxis, and cytokine binding (Koren et al., 1989; Krishna et al., 1997; Leroy et al., 2015).

Presently, in the United States, 103 million individuals reside in areas with unhealthy levels of O_3_ in ambient air [0.085–0.105 parts per million (ppm)], and globally, human exposure to O_3_ is increasing (American Lung Association, 2023; Malashock et al., 2022). Individuals with asthma, a chronic lung disease notably characterized by AHR, cough, dyspnea, persistent lung inflammation, and wheeze, are particularly sensitive to O_3_. Importantly, O_3_ is a non‐atopic asthma stimulus, and in furtherance of this, (1) emergency room visits for asthmatic children, adolescents, and adults increase as ambient O_3_ increases (Gharibi et al., 2019; Tolbert et al., 2000), (2) asthmatic children exhibit cough, shortness of breath, and heightened use of bronchodilators when ambient O_3_ is below the United States Environmental Protection Agency 8‐h primary and secondary National Ambient Air Quality Standards (0.070 ppm) (Gent et al., 2003), and (3) short‐term exposure to O_3_ is significantly associated with asthma mortality (Liu et al., 2019). Therefore, to prevent asthma morbidity and mortality that is associated with exposure to O_3_, it is important to understand cellular and molecular phenomena contributing to O_3_‐induced lung injury, lung inflammation, and AHR.

It is conceivable that chemerin‐like receptor 1 (CMKLR1) may limit the severity of O_3_‐induced asthma exacerbations. In humans (*Homo sapiens*) and mice (*Mus musculus*), CMKLR1 consists of 373 and 371 amino acids, respectively, and arises from translation of messenger ribonucleic acid (mRNA) generated from transcription of the chemerin chemokine‐like receptor 1 gene [*CMKLR1* (*Homo sapiens*); *Cmklr1* (*Mus musculus*)] (UniProt Consortium, 2023). CMKLR1 is a *G*_i/o_ protein‐coupled, seven‐transmembrane domain receptor with structural similarities to CC and CXC chemokine receptors and is expressed by adipocytes, epithelial cells, macrophages, monocytes, natural killer (NK) cells, myeloid and plasmacytoid dendritic cells, and vascular smooth muscle cells (Arita et al., 2005; Kennedy & Davenport, 2018; Kostopoulos et al., 2014; Luangsay et al., 2009; Parolini et al., 2007; Vermi et al., 2005). Chemotaxis of macrophages, NK cells, and myeloid and plasmacytoid dendritic cells are dependent, in part, on CMKLR1 (Luangsay et al., 2009; Parolini et al., 2007; Vermi et al., 2005), and each of these cells contribute to the pathogenesis of asthma (Gaurav & Agrawal, 2013; Gorska, 2017; van der Veen et al., 2020). At present, two endogenous ligands for CMKLR1 have been identified: resolvin E1 (RvE1) and chemerin. RvE1, a bioactive lipid, belongs to a class of molecules known as specialized pro‐resolving mediators while chemerin, a bioactive protein, is categorized as a non‐chemokine chemoattractant (Bondue, Wittamer, & Parmentier, 2011; Valente et al., 2022). Interestingly, both RvE1, a metabolite of the ω‐3 fatty acid, eicosapentaenoic acid, and chemerin, which is formed after proteolytic processing of its precursor, prochemerin, appear to be synthesized or enzymatically activated, respectively, within inflammatory exudates (Bondue, Wittamer, & Parmentier, 2011; Valente et al., 2022; Wittamer et al., 2005). Accordingly, both RvE1 and chemerin may contribute to inflammatory diseases, including asthma, via signaling through CMKLR1. Indeed, in mouse models of atopic asthma, intravenous administration of RvE1 or intranasal insufflation of chemerin diminished lung inflammation and increases in airway responsiveness while RvE1 also hastened the resolution of inflammation (Haworth et al., 2008, 2011; Zhao et al., 2014). Altogether, these data connote engagement of CMKLR1 with either of its ligands decreases the severity and enhances the resolution of atopic asthma.

Because exogenous administration of CMKLR1 ligands decreases the severity of antigen‐induced lung inflammation and AHR, we hypothesized that CMKLR1 limits the severity of lung pathophysiology induced by the non‐atopic asthma stimulus, O_3_. To test this hypothesis, phenotypic features of asthma were assessed in wild‐type mice and mice homozygous for a null mutation in the gene encoding *Cmklr1* (CMKLR1‐deficient mice) 4 and/or 24 h following cessation of a 3‐h acute inhalation exposure to either filtered room air (air) or O_3_.

## 2 MATERIALS AND METHODS

### 2.1 Animals

CMKLR1‐deficient mice [Mouse Genome Informatics (MGI) Identification: MGI:4430512] were generated via homologous recombination after replacing part of exon 3 of the *Cmklr1* gene with a β‐galactosidase (*lacZ*)‐aminoglycoside 3′‐phosphotransferase (*neo*) cassette (Luangsay et al., 2009; The Jackson Laboratory, 2023). CMKLR1‐deficient mice are viable, fertile, and exhibit no gross internal or external abnormalities.

We purchased four breeding pairs of mice heterozygous for a null mutation in *Cmklr1* (CMKLR1^+/−^ mice) from Deltagen, Inc. (San Mateo, CA, USA) via Charles River Laboratories (Wilmington, MA, USA). Male and female CMKLR1^+/−^ mice were mated to beget wild‐type, CMKLR1^+/−^, and CMKLR1‐deficient mice, which were genotyped via quantitative polymerase chain reaction (qPCR) at the *Cmklr1* allele by Transnetyx Inc. (Cordova, TN, USA) using deoxyribonucleic acid (DNA) extracted from tail snips. Male and female CMKLR1‐deficient mice, which were generated from the four breeding pairs of CMKLR1^+/−^ mice, procreated to produce CMKLR1‐deficient mice for this study. Because CMKLR1‐deficient mice were backcrossed into a C57BL/6NCrl genetic background for five generations, age‐matched C57BL/6NCrl mice (Strain Code 027; MGI Identification: MGI:2683688) were purchased from Charles River Laboratories at 7–8 weeks of age and used as wild‐type controls.

Under conditions we previously described (Razvi et al., 2015), all breeding pairs and their progeny were housed in the same room that was part of a multi‐species, modified barrier animal care facility. CMKLR1‐deficient mice were weaned at 21–27 days of age while wild‐type C57BL/6NCrl mice were acclimated to their new environment for at least 4 weeks prior to being subjected to any experimental procedures. Male mice were exclusively used in this study and entered an experimental protocol when at least 12 weeks of age.

### 2.2 Protocol

Mice, which were included in this study, were divided into three separate cohorts according to the experimental endpoint of interest. Nevertheless, regardless of their assigned cohort, all mice were acutely exposed via whole‐body inhalation to either air or O_3_ for 3 h. Mice in the first cohort were euthanized 4 or 24 h following cessation of exposure to either air or O_3_. Afterwards, blood was collected from, a bronchoalveolar lavage (BAL) performed on, and lungs excised from mice. Mice in the second cohort were anesthetized 24 h following cessation of exposure to either air or O_3_, and subsequently, respiratory system pressure‐volume (*P*–*V*) relationships and airway responsiveness to methacholine were determined. Mice in the final cohort were euthanized 24 h following cessation of exposure to air, and thereafter, lungs excised to measure wet and dry lung weights and calculate the lung wet‐to‐dry weight ratio.

### 2.3 Exposure to air or O_3_

Conscious mice were removed from their micro‐isolator cage (Tecniplast Group; Buguggiate, Varese, Lombardy, Italy), weighed, and immediately single‐housed in a cell [6.5″ (L) × 4.0″ (W) × 6.7″ (H)] that was part of a larger stainless steel wire mesh cage. After the wire mesh cage was placed inside a 75.5 L powder‐coated aluminum and Plexiglas® exposure chamber, the mice, without access to food (PicoLab Rodent Diet 20; LabDiet, Brentwood, MO, USA) or water, were acutely exposed via whole‐body inhalation to either air or O_3_ (2 ppm) for 3 h. Following cessation of the 3‐h exposure, the wire mesh cage was removed from the exposure chamber, and the mice were returned to their micro‐isolator cage where they once again had access to food and water ad libitum. Mice remained in their micro‐isolator cage until either 4 or 24 h following cessation of exposure. Additional details concerning these exposures were formerly provided (Razvi et al., 2015).

### 2.4 Blood collection and BAL

Four or twenty‐four hours following cessation of exposure to either air or O_3_, mice, which were part of the first cohort, were weighed and immediately euthanized with an intraperitoneal injection of pentobarbital sodium (200 mg/kg; Vortech Pharmaceuticals, Ltd., Dearborn, MI, USA). Subsequently, blood was collected from the heart, and the lungs were lavaged. An aliquot of whole blood was diluted in Turk blood diluting fluid (Ricca Chemical Company®; Arlington, TX, USA), and the total number of blood leukocytes enumerated with a hemacytometer (Hausser Scientific; Horsham, PA, USA). Via centrifugation, serum was isolated from whole blood and BAL fluid partitioned into liquid and cellular components. The total number of BAL cells was enumerated with a hemacytometer after resuspending the cell pellet in 1 mL of Hanks' balanced salt solution (HyClone Laboratories, Logan, UT, USA). BAL differential cell counts were accomplished by depositing an aliquot of resuspended BAL cells onto microscope slides, staining these slides with the Hema 3® stain set (Fisher Diagnostics; Middletown, VA, USA), and differentiating the cells based on standard morphological features using bright‐field microscopy (Fredrickson & Harris, 2000; Griesenbach et al., 2008). Adiponectin, chemerin, chitinase‐3‐like protein 1 (CHI3L1), hyaluronan, interleukin (IL)‐6, IL‐11, keratinocyte chemoattractant (KC), macrophage inflammatory protein (MIP)‐2, MIP‐3α, osteopontin, and receptor for advanced glycation end‐product (RAGE) were quantified in serum or BAL supernatant using Quantikine™ or DuoSet® enzyme‐linked immunosorbent assays from R&D Systems, Inc. (Minneapolis, MN, USA) while BAL albumin and protein were quantified, respectively, with an enzyme‐linked immunosorbent assay (Immunology Consultants Laboratory, Inc.; Portland, OR, USA) or a Pierce™ Bicinchoninic Acid (BCA) Protein Assay Kit from Thermo Fisher Scientific Inc. (Waltham, MA, USA). If a serum or BAL supernatant analyte was below the minimum detectable concentration of an immunoassay, we assigned the sample a value, which was calculated by dividing the minimum detectable concentration of the analyte by the square root of two (Hornung & Reed, 1990). More details concerning these methods can be found in Razvi et al. (2015).

### 2.5 Lung excision, RNA extraction, complementary deoxyribonucleic acid (cDNA) synthesis, and reverse transcription (RT)‐qPCR

All ensuing methods were formerly described in detail (Razvi et al., 2015), yet briefly, after bronchoalveolar lavages were complete, the animal's circulation was perfused with 10 mL of ice‐cold 1× phosphate‐buffered saline (PBS), and the left lung excised, snap‐frozen in liquid nitrogen, and stored at −80°C until needed. Subsequently, RNA was extracted from the lung lobe, and cDNA was synthesized from mRNA. Using cDNA, RT‐qPCR, and the comparative threshold cycle (*C*_T_) method (Livak & Schmittgen, 2001), the abundance of *Cmklr1* mRNA 4 or 24 h following cessation of exposure to O_3_ was expressed relative to the abundance of *Cmklr1* mRNA following cessation of exposure to air. All data were normalized to the abundance of glucuronidase, beta (*Gusb*) mRNA. Primers for both *Cmklr1* and *Gusb* were purchased from Bio‐Rad Laboratories, Inc. (Hercules, CA, USA).

### 2.6 Respiratory system *P*–*V* relationships and airway responsiveness to methacholine

Mice, which were part of the second cohort, were weighed, anesthetized, tracheostomized with a tubing adaptor, and with the assistance of a specialized ventilator (*flexiVent*; SCIREQ Scientific Respiratory Equipment Inc.; Montréal, Québec, Canada), artificially ventilated at a frequency of 2.5 Hz, a tidal volume of 0.3 mL, and a positive end‐expiratory pressure of 3 cm H_2_O 24 h following cessation of exposure to either air or O_3_. Additionally, the *flexiVent* was used to (1) deliver stepwise inspiratory and expiratory volume increments to generate respiratory system *P*–*V* relationships and (2) determine airway responsiveness to methacholine using the forced oscillation technique and constant phase model. From each *P*–*V* curve, quasistatic respiratory system elastance (*E*_stat_) was calculated. Total respiratory system impedance was partitioned into indices of airway responsiveness to methacholine [airway resistance (*R*_aw_), coefficient of lung tissue damping (*G*), and coefficient of lung tissue elastance (*H*)]. Methacholine was delivered to the animal in half‐logarithm intervals between 0.1 and 100 mg/mL. After airway responsiveness measurements were complete and while the animals remained under anesthesia, each mouse was euthanized by exsanguination after transecting the heart, which was accessed by performing a thoracotomy. Finally, more details concerning these methods have been previously described (Barreno et al., 2013; Malik et al., 2017).

### 2.7 Weight and dry lung weights and lung wet‐to‐dry weight ratio

Mice, which were part of the third cohort, were weighed and then euthanized with an intraperitoneal injection of sodium pentobarbital (200 mg/kg) 24 h following cessation of exposure to air. Subsequently, each mouse was subjected to a thoracotomy, which was followed by excision of the right and left lung lobes. After removing extraneous tissue from the lung lobes, the lobes were conjointly weighed on an analytical balance immediately after excision, and their mass was designated as the wet weight. Afterwards, the lobes were promptly placed in an oven for 5 days at 65°C. When 5 days had passed, the lobes were removed from the oven and conjointly weighed again on the same analytical balance. This mass was designated as the dry weight. The wet‐to‐dry weight ratio for the lungs was determined by dividing the wet weight by the dry weight.

### 2.8 Statistical analyses of data

The effect of genotype (wild‐type or CMKLR1‐deficient) and exposure (air or O_3_) on BAL or serum analytes was assessed by a two‐way analysis of variance (ANOVA) for normally distributed data or by a Kruskal–Wallis one‐way ANOVA for non‐normally distributed data. If the null hypothesis of the ANOVA was rejected, *post‐hoc* analysis following a two‐way ANOVA was performed using the Fisher's least significant difference (LSD) test while *post‐hoc* analysis following a Kruskal–Wallis one‐way ANOVA was performed using a Wilcoxon rank‐sum test. The relative abundance of *Cmklr1* mRNA was analyzed using a one‐way ANOVA. Body masses, total blood leukocytes, *E*_stat_, lung weights, and lung wet‐to‐dry weight ratios were compared using a Student's *t*‐test for unpaired samples. Prior to statistical analyses of methacholine dose–response curves with a three‐way mixed model repeated measures ANOVA (genotype × exposure × concentration of methacholine), responses to methacholine for *R*_aw_, *G*, and *H* were analyzed with a Shapiro–Wilk test for normality. If data did not conform to a normal distribution, data were analyzed further using a quantile‐quantile (q–q) plot. Since dose was significant across all treatments, pairwise *post‐hoc* comparisons were calculated with a Bonferroni *p*‐value adjustment for multiple comparisons. Methacholine dose–response curves were analyzed using R (R Core Team, 2023) while the remaining data were analyzed using JMP 16.1.0 (SAS Institute Inc.; Cary, NC, USA). Results are expressed as the means ± standard deviation. A *p* < 0.05 was considered significant.

## 3 RESULTS

### 3.1 Effect of CMKLR1 deficiency on body mass and total blood leukocytes

We observed no difference in body mass (31.5 ± 4.5 vs. 32.3 ± 3.7 g) between wild‐type and CMKLR1‐deficient mice, respectively, prior to commencement of exposure. In addition, the total number of blood leukocytes (3.4 ± 0.9 vs. 2.6 ± 0.9 × 10^6^/mL of blood) was not different between air‐exposed wild‐type and CMKLR1‐deficient mice, respectively.

### 3.2 Effect of O_3_ on the relative abundance of lung *Cmklr1* mRNA in wild‐type mice

**FIGURE 1:**
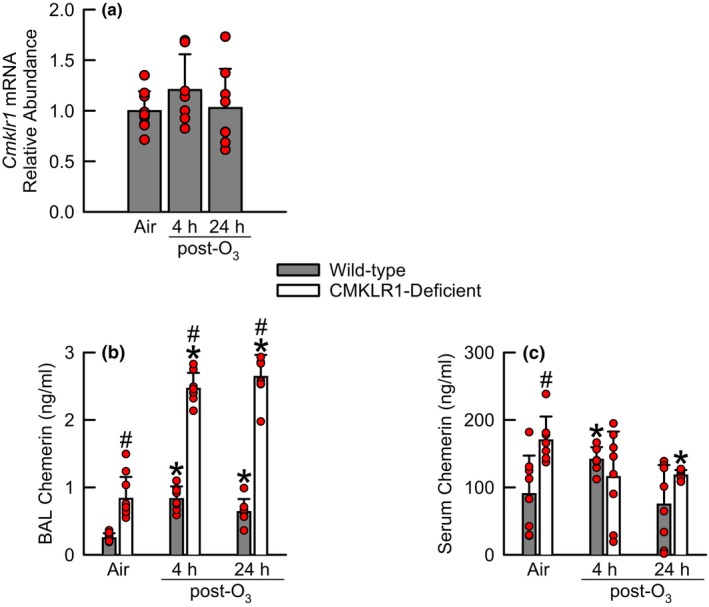
(a) Relative abundance of chemerin chemokine‐like receptor 1 (*Cmklr1*) messenger ribonucleic acid (mRNA) in the left lung lobe obtained from wild‐type C57BL/6NCrl mice and concentration of chemerin in (b) bronchoalveolar lavage (BAL) fluid and (c) serum of wild‐type C57BL/6NCrl mice or mice genetically deficient in the gene encoding *Cmklr1* (CMKLR1‐deficient mice) 4 or 24 h following cessation of a 3‐h exposure to either filtered room air (air) or ozone (O_3_; two parts per million). *Cmklr1* mRNA relative abundance was analyzed via a one‐way analysis of variance (ANOVA). BAL chemerin was analyzed via a Kruskal–Wallis one‐way ANOVA while subsequent pairwise *post‐hoc* comparisons were calculated using a Wilcoxon rank‐sum test. Serum chemerin was analyzed via a two‐way ANOVA, and pairwise *post‐hoc* comparisons were calculated using a Fisher's least significance difference test. Each value is expressed as the mean ± standard deviation. *n* = 7–10 mice for each group. *p < 0.05 compared to genotype‐matched mice exposed to air. ^#^ *p* < 0.05 compared to wild‐type mice subjected to an identical exposure.

When expressed relative to *Cmklr1* mRNA from air‐exposed wild‐type mice, the relative abundance of *Cmklr1* mRNA was unaltered by exposure to O_3_ in wild‐type mice either 4 or 24 h following cessation of exposure (Figure 1a).

### 3.3 Effect of CMKLR1 deficiency and O_3_ on BAL and serum chemerin

As illustrated in Figure 1b, chemerin was detectable in BAL fluid obtained from air‐exposed wild‐type and CMKLR1‐deficient mice. However, BAL chemerin was three times greater in CMKLR1‐deficient as compared to wild‐type mice. Irrespective of genotype or the time following cessation of exposure, BAL chemerin was significantly greater in O_3_‐ as compared to air‐exposed mice (Figure 1b). Nevertheless, at 4 and 24 h following cessation of exposure to O_3_, BAL chemerin was, on average, three and a half times greater in CMKLR1‐deficient as compared to wild‐type mice.

Chemerin was detectable in serum isolated from blood of wild‐type and CMKLR1‐deficient mice, and in air‐exposed mice, serum chemerin was two times greater in CMKLR1‐deficient as compared to wild‐type mice (Figure 1c). When compared to genotype‐matched, air‐exposed controls, serum chemerin was increased in wild‐type mice 4‐h following cessation of exposure to O_3_ while decreased in CMKLR1‐deficient mice 24 h following cessation of O_3_ exposure.

### 3.4 Effect of CMKLR1 deficiency on O_3_‐induced lung injury

**FIGURE 2:**
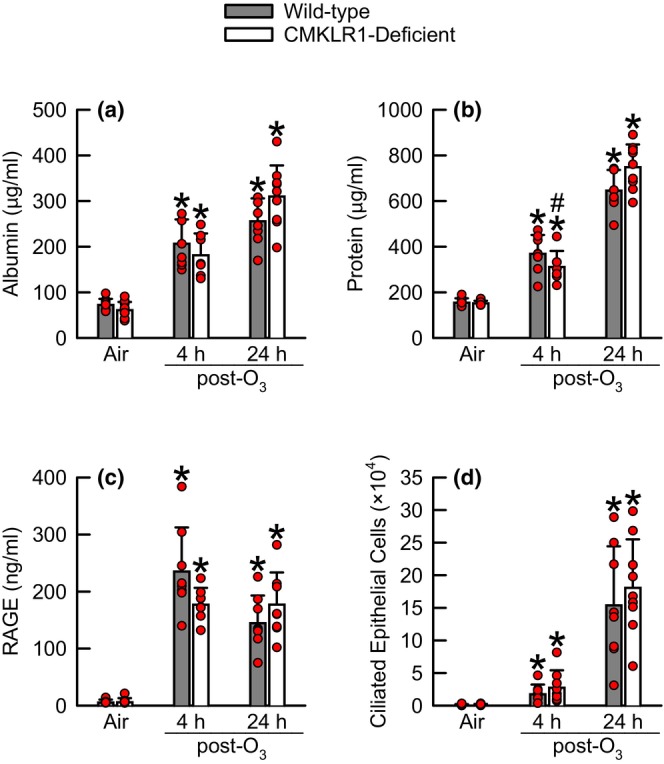
Concentration of bronchoalveolar lavage (BAL) fluid (a) albumin, (b) protein, and (c) receptor for advanced glycation end‐product (RAGE) in addition to the number of (d) BAL ciliated epithelial cells obtained from wild‐type C57BL/6NCrl mice or mice genetically deficient in the gene encoding *Cmklr1* (CMKLR1‐deficient mice) 4 or 24 h following cessation of a 3‐h exposure to either filtered room air (air) or ozone (O_3_; two parts per million). All data were analyzed via a two‐way analysis of variance (ANOVA) and pairwise *post‐hoc* comparisons were calculated using a Fisher's least significance difference test. Each value is expressed as the mean ± standard deviation. *n* = 7–10 mice for each group. *p < 0.05 compared to genotype‐matched mice exposed to air. ^#^ *p* < 0.05 compared to wild‐type mice subjected to an identical exposure.

O_3_‐induced lung injury was assessed by quantifying BAL albumin and protein, indices of lung permeability, BAL RAGE, an indice of alveolar epithelial type I cell damage, and BAL ciliated epithelial cells, an indice of epithelial cell desquamation (Figure 2); (Bhalla, 1999; McElroy & Kasper, 2004; Scheel et al., 1959). No genotype‐related differences in any indice existed following exposure to air. As compared to genotype‐matched, air‐exposed controls, BAL albumin, protein, RAGE, and ciliated epithelial cells were significantly greater following O_3_ exposure. Apart from BAL protein, which was significantly lower in CMKLR1‐deficient as compared to wild‐type mice 4 h following cessation of O_3_ exposure, no other genotype‐related differences existed between wild‐type and CMKLR1‐deficient mice.

### 3.5 Effect of CMKLR1 deficiency on O_3_‐induced lung inflammation

**FIGURE 3:**
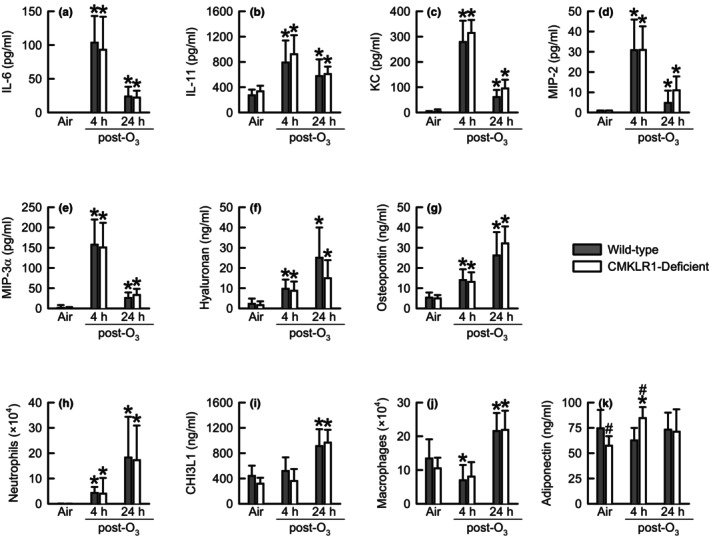
Bronchoalveolar lavage (BAL) fluid (a) interleukin (IL)‐6, (b) IL‐11, (c) keratinocyte chemoattractant (KC), (d) macrophage inflammatory protein (MIP)‐2, (e) MIP‐3α, (f) hyaluronan, (g) osteopontin, (h) neutrophils, (i) chitinase‐3‐like protein 1 (CHI3L1), (j) macrophages, and (k) adiponectin obtained from wild‐type C57BL/6NCrl mice or mice genetically deficient in the gene encoding *Cmklr1* (CMKLR1‐deficient mice) 4 or 24 h following cessation of a 3‐h exposure to either filtered room air (air) or ozone (O_3_; two parts per million). IL‐6, KC, MIP‐2, MIP‐3α, neutrophils, and adiponectin were analyzed via a Kruskal–Wallis one‐way analysis of variance (ANOVA) while subsequent pairwise *post‐hoc* comparisons were calculated using a Wilcoxon rank‐sum test. IL‐11, hyaluronan, osteopontin, CHI3L1, and macrophages were analyzed via a two‐way ANOVA and pairwise *post‐hoc* comparisons were calculated using a Fisher's least significance difference test. Each value is expressed as the mean ± standard deviation. *n* = 7–10 mice for each group. *p < 0.05 compared to genotype‐matched mice exposed to air. ^#^ *p* < 0.05 compared to wild‐type mice subjected to an identical exposure.

To determine if CMKLR1 contributed to the development of lung inflammation induced by acute inhalation exposure to O_3_, we quantified BAL moieties (adiponectin, CHI3L1, hyaluronan, IL‐6, IL‐11, KC, MIP‐2, MIP‐3α, and osteopontin) and leukocytes (macrophages and neutrophils), which influence the course of oxidant‐induced lung pathophysiology Figure 3; (Barreno et al., 2013; DeLorme et al., 2002; Garantziotis et al., 2016; Johnston et al., 2022; Johnston, Mizgerd, & Shore, 2005; Johnston, Schwartzman, et al., 2005; Mathews et al., 2014; Pendino et al., 1995; Sohn et al., 2010; Sue et al., 2004; Ward et al., 2000; Waxman et al., 1998; Zhu et al., 2010). Apart from adiponectin (Figure 3k), which was significantly lower in CMKLR1‐deficient as compared to wild‐type mice, no differences in any other indice existed following cessation of exposure to air. As compared to genotype‐matched, air‐exposed controls, exposure to O_3_ significantly increased IL‐6, IL‐11, KC, MIP‐2, MIP‐3α, hyaluronan, osteopontin, neutrophils, CHI3L1, and macrophages (Figure 3a–j). However, these increases were dependent upon the time elapsed following cessation of O_3_ exposure. Nevertheless, no genotype‐related differences in BAL IL‐6, IL‐11, KC, MIP‐2, MIP‐3α, hyaluronan, osteopontin, neutrophils, CHI3L1, or macrophages were extant following O_3_ exposure. At 4 h following cessation of exposure, adiponectin was significantly greater in O_3_‐ as compared to air‐exposed CMKLR1‐deficient mice (Figure 3k). Additionally, at this time, adiponectin was greater in O_3_‐exposed CMKLR1‐deficient as compared to O_3_‐exposed wild‐type mice. Finally, irrespective of genotype or exposure, eosinophils and lymphocytes were rarely recovered from lungs of mice subjected to BAL (data not shown).

### 3.6 Effect of CMKLR1 deficiency on O_3_‐induced airway hyperresponsiveness

**FIGURE 4:**
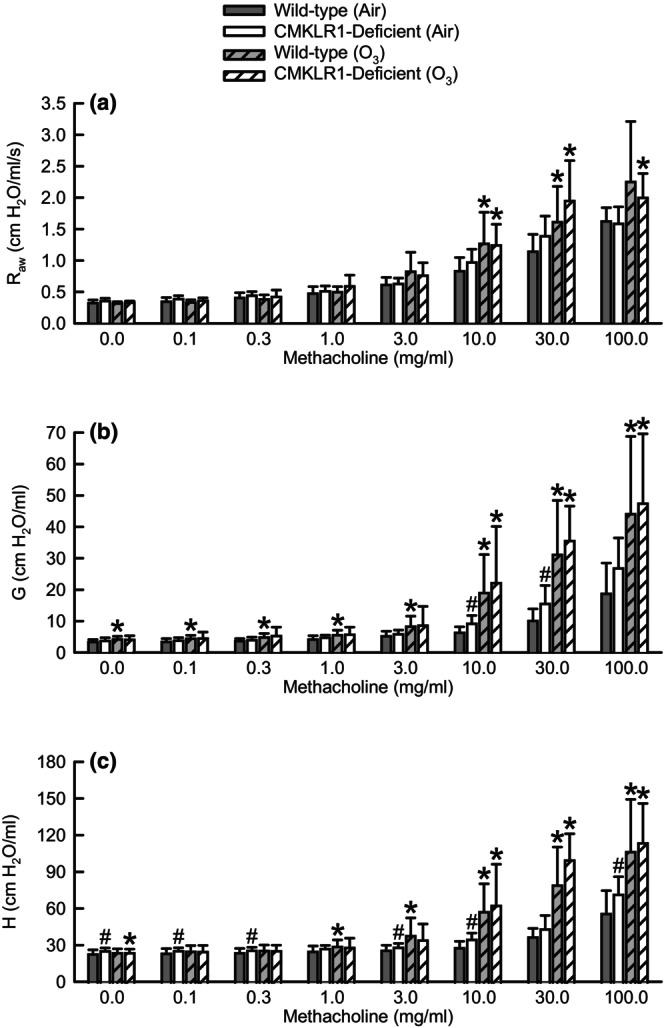
Responses to aerosolized acetyl‐β‐methylcholine chloride (methacholine) for (a) airway resistance (*R*_aw_), (b) coefficient of lung tissue damping (*G*), and (c) coefficient of lung tissue elastance (*H*) from wild‐type C57BL/6NCrl mice or mice genetically deficient in the gene encoding *Cmklr1* (CMKLR1‐deficient mice) 24 h following cessation of a 3‐h exposure to either filtered room air (air) or ozone (O_3_; two parts per million). Dose–response curves were analyzed via a three‐way mixed model repeated measures analysis of variance, and pairwise *post‐hoc* comparisons were calculated with a Bonferroni *p*‐value adjustment for multiple comparisons. Each value is expressed as the mean ± standard deviation. *n* = 8–11 mice for each group. *p < 0.05 compared to genotype‐matched mice exposed to air. ^#^ *p* < 0.05 compared to wild‐type mice subjected to an identical exposure.

Twenty‐four hours following cessation of exposure to air, basal *H* was significantly greater in CMKLR1‐deficient as compared to wild‐type mice while there was no effect of genotype on basal *R*_aw_ or *G* (Table 1; Figure 4a–c). Compared to genotype‐matched, air‐exposed controls, basal *G* was significantly increased while basal *H* was significantly decreased following O_3_ exposure in wild‐type and CMKLR1‐deficient mice, respectively (Table 1; Figure 4b,c).

**TABLE 1:** Basal airway and lung parenchymal oscillation mechanics for wild‐type and CMKLR1‐deficient mice exposed to either filtered room air or ozone.

Genotype (exposure)	*R*_aw_ (cm H_2_O/mL/s)	*G* (cm H_2_O/mL)	*H* (cm H_2_O/mL)
Wild‐type (air)	0.33 ± 0.04	3.75 ± 0.40	23.86 ± 2.27
CMKLR1‐deficient (air)	0.36 ± 0.04	4.10 ± 0.58	26.41 ± 1.25**
Wild‐type (O_3_)	0.33 ± 0.02	4.54 ± 0.60*	24.88 ± 2.04
CMKLR1‐deficient (O_3_)	0.34 ± 0.02	4.42 ± 0.92	24.93 ± 1.73*

*Note*: Measurements of basal airway (*R*_aw_) and lung parenchymal (*G* and *H*) oscillation mechanics were made following administration of aerosolized 1× phosphate‐buffered saline 24 h following cessation of a three‐hour exposure to either air or O_3_. The results are expressed as the mean ± the standard deviation. *n* = 8–11 mice in each group.

Abbreviations: air, filtered room air; CMKLR1, chemerin‐like receptor 1; *G*, coefficient of lung tissue damping; *H*, coefficient of lung tissue elastance; O_3_, ozone; *R*_aw_, airway resistance.

* *p* < 0.05 compared to genotype‐matched mice exposed to air.

** *p* < 0.05 compared to wild‐type mice subjected to an identical exposure.

Regardless of genotype or exposure, methacholine significantly increased responses to *R*_aw_, *G*, and *H*. However, following exposure to air, responses to methacholine for *G* (10 and 30 mg/mL) and *H* (0.1, 0.3, 3, 10, and 100 mg/mL) were significantly greater in CMKLR1‐deficient as compared to wild‐type mice (Figure 4b,c). As compared to genotype‐matched, air‐exposed controls, exposure to O_3_ enhanced responses to methacholine for *R*_aw_, *G*, and *H* (Figure 4a–c). Nevertheless, there were no genotype‐related differences in any indice following O_3_ exposure.

### 3.7 Effect of CMKLR1 deficiency on quasi‐static respiratory system *P*–*V* relationships

**FIGURE 5:**
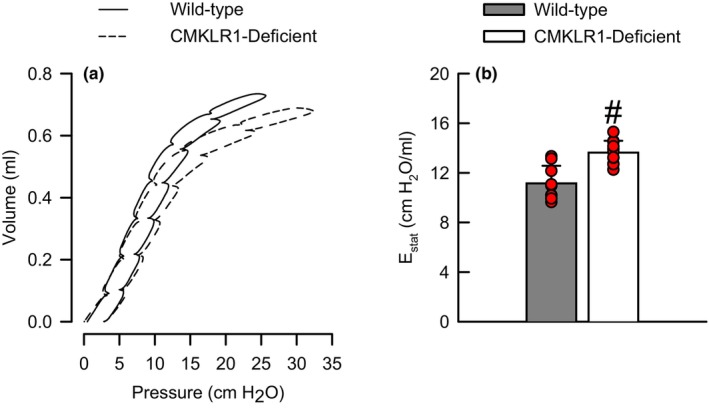
(a) Quasi‐static respiratory system pressure‐volume curves and (b) quasi‐static respiratory system elastance (*E*_stat_) obtained from wild‐type C57BL/6NCrl mice or mice genetically deficient in the gene encoding *Cmklr1* (CMKLR1‐deficient mice) 24 h following cessation of a 3‐h exposure to filtered room air. *E*_stat_ data were compared using a Student's *t*‐test for unpaired samples and are expressed as the mean ± the standard deviation. *n* = 9–11 mice for each group. ^#^ *p* < 0.05 compared to wild‐type mice.

Since we observed differences in basal *H* between air‐exposed wild‐type and CMKLR1‐deficient mice (Table 1; Figure 4c), we determined whether this was reflective of “true” changes in the elastic properties of the respiratory system. To that end, we generated quasistatic *P*–*V* curves in air‐exposed wild‐type and CMKLR1‐deficient mice. As illustrated in Figure 5a, *P*–*V* curves of CMKLR1‐deficient mice were shifted to the right of wild‐type mice while quasi‐static respiratory system elastance (*E*_stat_) measured over the deflation portion of the *P*–*V* curve was significantly greater in CMKLR1‐deficient as compared to wild‐type mice (Figure 5b).

### 3.8 Effect of CMKLR1 deficiency on lung weight and lung wet‐to‐dry weight ratio

Lung size is an important determinant of lung compliance, and hence, lung elastance (Beachey, 2023). Thus, to determine if genotype‐related differences in *E*_stat_ were a result of differences in lung size (Figure 5b), we measured the wet and dry weights of lungs excised from air‐exposed wild‐type and CMKLR1‐deficient mice. In addition, from these data, we calculated the lung wet‐to‐dry weight ratios. As shown in Table 2, we observed no genotype‐related differences in either the wet or dry lung weights or the lung wet‐to‐dry weight ratios.

**TABLE 2:** Wet and dry lung weights and lung wet‐to‐dry weight ratios for wild‐type and CMKLR1‐deficient mice exposed to filtered room air.

Genotype	Wet weight (g)	Dry weight (g)	Wet‐to‐dry weight ratio
Wild‐type	0.133 ± 0.004	0.032 ± 0.001	4.09 ± 0.11
CMKLR1‐deficient	0.132 ± 0.003	0.031 ± 0.001	4.24 ± 0.15

*Note*: Lungs were excised from animals 24 h following cessation of a 3‐h exposure to filtered room air. The results are expressed as the mean ± the standard deviation. *n* = 5 mice in each group.

Abbreviation: CMKLR1, chemerin‐like receptor 1.

## 4 DISCUSSION

Contrary to our hypothesis, we uncovered no evidence to asseverate crucial contributions from CMKLR1 to pathological lung sequelae induced by acute inhalation exposure to O_3_, including lung hyperpermeability, neutrophil migration to air spaces, and AHR. However, in the absence of any inciting stimulus, we demonstrate CMKLR1 enables the course of innate airway responsiveness and lung elastic recoil.

Methner et al. (1997) were first to report expression of *Cmklr1* in organs of adult mice, including brain, lung, parathyroid gland, and thymus. Consistent with Methner et al. (1997) and other investigators (Demoor et al., 2011; Kilburg‐Basnyat et al., 2018; Luangsay et al., 2009), we also demonstrate expression of *Cmklr1* in lungs obtained from adult mice (Figure 1a), yet O_3_ did not alter *Cmklr1* expression either 4 or 24 h following cessation of exposure. In contrast, Kilburg‐Basnyat et al. (2018) reported lung *Cmklr1* expression was significantly decreased in male C57BL/6J mice 24 h following cessation a 3‐h exposure to O_3_ (1 ppm). However, there are at least three potential explanations for the discrepancy between our results and those of Kilburg‐Basnyat et al. (2018). First, we determined *Cmklr1* expression in lungs harvested from C57BL/6NCrl mice from Charles River Laboratories, Inc. while Kilburg‐Basnyat et al. (2018) used lungs excised from C57BL/6J mice from The Jackson Laboratory. Divergent behavioral, cardiac, and immune phenotypes exist among these two substrains as a probable result of genetic differences (Fischer et al., 2019; Garcia‐Menendez et al., 2013; Khisti et al., 2006; Zurita et al., 2011). Therefore, following exposure to O_3_, it is plausible that disparity in lung *Cmklr1* mRNA expression among these two substrains is a result of genetic differences. Second, the effect of O_3_ on breathing patterns is concentration dependent. For example, breathing patterns [minute ventilation (*V̇*_E_), breathing frequency, and tidal volume] are suppressed to a greater extent in mice exposed to 2 as compared to 1 ppm O_3_ for 3 h (Shore et al., 2002). Because the dose of O_3_ delivered to the lungs is the product of O_3_ concentration, exposure time, and *V̇*_E_ (Wiester et al., 1987), it is conceivable that the dose of O_3_ delivered to the lungs of the mice in these two studies is different, which may impact gene expression. Third, our mice were at least 12 weeks of age at the time of study while those in the study of Kilburg‐Basnyat et al. were between 8 and 12 weeks of age (Kilburg‐Basnyat et al., 2018). Differences exist between 7‐ and 39‐week‐old mice with regard to O_3_‐lung inflammation and anti‐oxidant gene expression (Shore et al., 2011), and thus, age is another plausible explanation as to the reason differences in lung *Cmklr1* mRNA expression exist between our study and that of Kilburg‐Basnyat et al. (2018).

Regardless of genotype, chemerin, one of two endogenous ligands for CMKLR1, was significantly greater in BAL fluid obtained from O_3_‐ as compared to air‐exposed mice (Figure 1b), which is consistent with our previous data (Malik et al., 2017; Razvi et al., 2015). However, independent of exposure, BAL chemerin was at least three‐fold greater in CMKLR1‐deficient as compared to wild‐type mice, and this observation is not without precedent. First, Bondue, Vosters, et al. (2011) reported that BAL chemerin was greater in CMKLR1‐deficient as compared to wild‐type mice, which were both infected with the pneumonia virus of mice (PMV). Second, Provoost et al. (2016) reported BAL chemerin was greater in CMKLR1‐deficient as compared to wild‐type mice exposed to diesel exhaust particles, house dust mite, or the combination of diesel exhaust particles and house dust mite. High affinity engagement of chemerin with CMKLR1 leads to activation of intracellular signaling cascades and internalization of the chemerin‐CMKLR1 receptor complex (Bondue, Wittamer, & Parmentier, 2011). Therefore, it is plausible that BAL chemerin is increased in CMKLR1‐deficient mice because chemerin cannot be efficiently internalized in the absence of CMKLR1. Serum chemerin was also greater in CMKLR1‐deficient as compared to wild‐type mice following exposure to air (Figure 1c). However, the loss of CMKLR1 in O_3_‐exposed mice had variable effects on serum chemerin (Figure 1c), which could imply that internalization of chemerin via CMKLR1 in the circulation, following exposure to O_3_, is not as prevalent or as efficient as it is in the epithelial lining fluid of the lung. Although we did not measure BAL or serum RvE1, it would be of interest to determine if BAL and serum RvE1 levels are altered in the absence of CMKLR1.

To date, four publications have described lung inflammation in CMKLR1‐deficient mice, which resulted from exposure to diverse inciting stimuli, including cigarette smoke, diesel exhaust particles, house dust mite, lipopolysaccharide (LPS), and PMV (Bondue, Wittamer, & Parmentier, 2011; Demoor et al., 2011; Luangsay et al., 2009; Provoost et al., 2016). In these studies, which investigators quantified BAL chemerin in wild‐type mice (Bondue, Wittamer, & Parmentier, 2011; Demoor et al., 2011; Provoost et al., 2016), each inflammatory stimulus increased chemerin except for house dust mite. However, depending upon the (1) inflammatory stimulus and (2) time BAL fluid and lung tissue were harvested from mice after cessation of exposure, data demonstrate that the presence of CMKLR1 can either increase or decrease inflammatory indices. In Table 3, we summarize the effect of CMKLR1 deficiency on inflammatory indices pertinent to our study. From these data, it is evident that inflammatory cell recruitment to the air spaces and lungs and appearance of inflammatory cytokines in BAL fluid and lung tissue are highly dependent upon the inflammatory stimulus, which could prompt different host responses and subsequent involvement of CMKLR1. Similar phenomena have been reported in mice deficient in receptors with structural similarities to CMKLR1. For example, chemokine (C‐C) motif receptor (Ccr) 2 (Ccr2) limits the severity of lung inflammation in a mouse model of fungal asthma but contributes to expression of pro‐inflammatory cytokines following chronic exposure to LPS (Blease et al., 2000; Cui et al., 2020). In addition, chemokine (C‐C) motif receptor‐like 2 (Ccrl2), a chemerin receptor with no signaling capabilities, has no effect on lung inflammation induced by exposure to O_3_ but facilitates inflammatory processes in a mouse model of atopic asthma (Malik et al., 2017; Otero et al., 2010). Nevertheless, we were surprised that no genotype‐related differences in lung injury and lung inflammation were observed following acute inhalation exposure to O_3_ (Figures 2 and 3).

**TABLE 3:** Effect of genetic deficiency of CMKLR1 in mice on indices of lung inflammation in response to inflammatory stimuli.^a^

Study	Inflammatory stimulus	IL‐6	KC	Neutrophils	Macrophages
Bondue, Vosters, et al. (2011)	Pneumonia virus of mice	Greater than wild‐type	Greater than wild‐type	Greater than wild‐type	Greater than wild‐type
Demoor et al. (2011)	Cigarette smoke	−^b^	Less than wild‐type	Less than wild‐type	−
Luangsay et al. (2009)	Lipopolysaccharide	No effect	No effect	Greater than wild‐type	Greater than wild‐type
Provoost et al. (2016)	Diesel exhaust particles	−	−	No effect	No effect
Provoost et al. (2016)	House dust mite	−	−	No effect	−
Provoost et al. (2016)	Diesel exhaust particles + house dust mite	−	−	Greater than wild‐type	−

Abbreviations: CMKLR1, chemerin‐like receptor 1; IL‐6, interleukin‐6; KC, keratinocyte chemoattractant.

^a^ Qualitative comparisons of bronchoalveolar lavage or lung tissue inflammatory indices were made between wild‐type and CMKLR1‐deficient mice following exposure or conjoint exposure to inflammatory stimuli.

^b^ Indice was not measured.

Emblematic features of lung injury (i.e., lung hyperpermeability and ciliated airway epithelial cell desquamation) and lung inflammation (i.e., neutrophil migration to air spaces) induced by inhalation of O_3_ are observed in humans and mice (Johnston et al., 2022; Koren et al., 1989; Krishna et al., 1997; Malik et al., 2017). We and other investigators have reported that manifestation of these features is mechanistically coupled to inflammatory cytokines or their receptors. For example, neutrophil migration to the air spaces following O_3_ exposure is dependent upon adiponectin, IL‐6, osteopontin, and chemokine (C‐X‐C motif) receptor 2, which is the receptor for KC and MIP‐2 (Barreno et al., 2013; Johnston, Mizgerd, & Shore, 2005; Johnston, Schwartzman, et al., 2005; Zhu et al., 2010). In wild‐type and CMKLR1‐deficient mice infected with PMV, IL‐6 and KC in lung tissue homogenates were increased to a greater extent in CMKLR1‐deficient as compared to wild‐type mice concurrently with BAL neutrophils [Table 3 and (Bondue, Vosters, et al., 2011)]. Alternatively, BAL KC and neutrophils were concomitantly decreased to a greater extent in CMKLR1‐deficient as compared to wild‐type mice following chronic exposure to cigarette smoke Table 3; (Demoor et al., 2011). Neither IL‐6 nor KC were different between wild‐type and CMKLR1‐deficient mice following cessation of O_3_ exposure (Figure 3a,c). Furthermore, MIP‐2 and osteopontin were unaffected by CMKLR1 deficiency (Figure 3d,g), yet BAL adiponectin was significantly greater in O_3_‐exposed CMKLR1‐deficient as compared to O_3_‐exposed wild‐type mice 4 h following cessation of exposure (Figure 3k). Nevertheless, this difference was small. Consequently, it is not surprising that any genotype‐related differences in BAL neutrophils were extant following O_3_ exposure (Figure 3h). Adiponectin and IL‐6 influence O_3_‐induced lung hyperpermeability while IL‐6 and CXCR2 contribute to desquamation of ciliated airway epithelial cells (Johnston, Mizgerd, & Shore, 2005; Lang et al., 2008; Zhu et al., 2010). Nevertheless, adiponectin, IL‐6, KC, and MIP‐2 were either minorly affected or not affected by CMKLR1 deficiency following O_3_ exposure, which is consistent with our observation of no genotype‐related differences in lung hyperpermeability (i.e., BAL albumin and protein) (Figure 2a,b) or BAL ciliated epithelial cells (Figure 2d).

We considered at least three explanations as to the reason CMKLR1 deficiency influences lung inflammation in response to some stimuli but not others. First, as we discussed in the fourth paragraph of the Discussion, data clearly demonstrate that the contribution of CMKLR1 to lung inflammation is stimulus specific. Second, for each of the studies in Table 3, mice were either exposed to an inflammatory stimulus on a single occasion (PMV or LPS) or repeatedly (cigarette smoke, diesel exhaust particles, and house dust mite) (Bondue, Vosters, et al., 2011; Demoor et al., 2011; Luangsay et al., 2009; Provoost et al., 2016). Nevertheless, although PMV or LPS were administered on a single occasion, these stimuli remained present until the animals were euthanized whereas in this study mice were acutely exposed to O_3_ on a single occasion and then promptly removed from any further exposure to the inciting stimulus. Consequently, it is plausible that the contribution of CMKLR1 to lung injury and lung inflammation is not only dependent upon the identity of inflammatory stimulus but also upon duration of exposure. Evidence for the latter possibility is supported by data from Kleeberger et al. (Kleeberger et al., 1993) who demonstrated that separate genetic loci control neutrophil migration to the air spaces following acute (2 ppm for 3 h) as compared to subacute (0.3 ppm for 48 h) exposure to O_3_. These data foster the presumption that CMKLR1 may impact the development of lung injury and lung inflammation following continuous and prolonged exposure to O_3_. Third, in the absence of CMKLR1, more chemerin is available for engagement with chemerin‐like receptor 2 (CMKLR2), which is a second G protein‐coupled receptor for chemerin that can activate signal transduction cascades (Bondue, Wittamer, & Parmentier, 2011). Thus, if CMKLR2 exerts the opposite effect on O_3_‐induced lung injury and lung inflammation than that which occurs when CMKLR1 is absent, no net effect on any indice would manifest in CMKLR1‐deficient mice.

In the absence of any inciting stimulus, basal *H*, a measure of lung tissue elastance, was significantly greater in CMKLR1‐deficient as compared to wild‐type mice [Table 1; Figure 4c, and (Bates et al., 2011)]. Consistent with these data, respiratory system *P*–*V* curves generated from air‐exposed CMKLR1‐deficient mice were shifted to the right and *E*_stat_ was significantly greater in CMKLR1‐deficient as compared to wild‐type mice (Figure 5). Conjointly, these data demonstrate that elastic recoil is increased in CMKLR1‐deficient mice in the absence of any inciting stimulus, and we considered several scenarios, which could explain this phenomenon. First, CMKLR1 signaling stimulates cell proliferation and migration (Kaur et al., 2010; Liu et al., 2016), and thus, the absence of CMKLR1 could impede growth of tissues and organs, including the lungs. Thus, we contemplated whether differences in lung size could account for genotype‐related differences in elastic recoil since compliance is decreased, and hence, elastance increased in smaller lungs (Beachey, 2023; Lu et al., 2006). Nevertheless, our data do not support this scenario since neither wet nor dry lung weights were affected by genotype in air‐exposed mice (Table 2). Second, the presence of lung edema in CMKLR1‐deficient mice resulting from a compromised alveolar‐capillary membrane could increase lung stiffness since gas is displaced by fluid in alveoli and small airways (Parker & Townsley, 2004). However, this does not seem plausible since we observed no genotype‐related differences in BAL protein or albumin or the lung wet‐to‐dry weight ratio between air‐exposed wild‐type and CMKLR1‐deficient mice (Figure 2a,b; Table 2). If lung edema was the basis for greater elastic recoil in CMKLR1‐deficient mice, we would expect each of these indices to be greater in CMKLR1‐deficient mice (Matute‐Bello et al., 2011). Third, exaggerated airway closure, which could arise from greater airway constriction or instability of the peripheral airways (Heil et al., 2008; Wagers et al., 2007), in CMKLR1‐deficient mice could account for increased elastic recoil. It is doubtful that greater airway constriction leads to enhanced airway closure in CMKLR1‐deficient mice since there were no differences in baseline *R*_aw_ between air‐exposed wild‐type and CMKLR1‐deficient mice (Table 1; Figure 4a). Alternatively, lung surfactant is an important determinant of peripheral airway stability, and accordingly, airway closure (Heil et al., 2008). Zhao et al. (2016) demonstrated that exogenous administration of RvD1 was able to restore expression of surfactant protein A (SP‐A), which was significantly decreased in a rat model of ischemia–reperfusion lung injury. Since RvE1, a CMKLR1 ligand, and RvD1 belong to the same family of lipid‐derived pro‐resolving mediators, it is tempting to speculate that RvE1‐CMKLR1 signaling could also impact expression of lung surfactant. If true, loss of CMKLR1 would decrease surfactant expression, increase instability of peripheral airways, and enhance airway closure. Nevertheless, additional experiments are needed to confirm this line of thought. Fourth and finally, differences in the arrangement of collagen fibers in the lungs of wild‐type and CMKLR1‐deficient mice could explain genotype‐related differences in elastic recoil. For example, in contrast to the relatively heterogenous alignment of collagen fibers in healthy lungs, fibrotic lungs consist of dense, highly aligned collagen fibers (Tisler et al., 2020). Cash et al. (2014) demonstrate via a computer algorithm that a CMKLR1 ligand decreases collagen alignment, which would theoretically decrease lung elastance. Consequently, the absence of CMKLR1 could lead to greater alignment of collagen fibers, and thus, increased lung stiffness. Regardless of the mechanism, this is the first study to demonstrate that the lungs of CMKLR1‐deficient mice are intrinsically stiffer in the absence of any inciting stimulus.

We also observed that CMKLR1 limits the severity of innate airway responsiveness to methacholine in the absence of any inciting stimulus (Figure 4b, c). Using the constant phase model, we determined that the ability of CMKLR1 to reduce the severity of innate airway responsiveness is localized to the lung parenchyma, which is represented by *G* and *H* (Figure 4b, c), and not the central airways since *R*_aw_ is not different between air‐exposed wild‐type and CMKLR1‐deficient mice (Figure 4a). Among responses to methacholine for *G* and *H*, differences in *H*, a measure of lung elastance, were the most pronounced between genotypes following cessation of exposure to air [Figure 4c and (Bates et al., 2011)], and we considered four potential possibilities for this phenomenon. First, enhanced airway closure due to greater airway constriction could increase responses to methacholine for *H* (Wagers et al., 2004, 2007). However, this is not probable since there were no genotype‐related differences in *R*_aw_ (Figure 4a). Second, differences in the gut microbiome impact the development of airway responsiveness (Cho et al., 2018), and Dranse et al. (2018) demonstrated that wild‐type and CMKLR1‐deficient mice housed in the same facility exhibited significant differences in the abundance of various bacterial taxa in their gut. Thus, if present in our mice, genotype‐related differences in the gut microbiome could explain differences in innate airway responsiveness. Third, increased responses to methacholine for *H* could manifest in CMKLR1‐deficient mice consequent to surfactant dysfunction or loss. Fourth, it is also possible that CMKLR1 deficiency alters the lung anatomy in such a manner as to increase airway responsiveness.

Although O_3_ significantly increased airway responsiveness to methacholine in mice of both genotypes as compared to genotype‐matched, air‐exposed controls, genotype‐related differences in airway responsiveness observed in mice exposed to air were completely abolished (Figure 4b, c). O_3_ deactivates lung surfactant (Podgorski et al., 2001), which would increase responses to methacholine for H because of greater instability of the peripheral airways (Heil et al., 2008). If surfactant is maximally deactivated by CMKLR1 deficiency alone, it is plausible that exposure to O_3_ may not cause further deactivation that results in greater responses to methacholine for *H* in O_3_‐exposed CMKLR1‐deficient mice. Additionally, we found no genotype‐related differences among O_3_‐exposed mice for BAL hyaluronan, IL‐11, KC, MIP‐2, and osteopontin, which promote O_3_‐induced AHR [(Figure 3b–d,f,g) and (Barreno et al., 2013; Garantziotis et al., 2016; Johnston et al., 2022; Johnston, Mizgerd, & Shore, 2005)]. Thus, this is an additional rationale as to the reason no genotype‐related differences in airway responsiveness were observed following exposure to O_3_. Nevertheless, these data demonstrate that CMKLR1 has a significant impact on the contribution of the lung parenchyma to innate airway responsiveness.

In summary, we demonstrate that failure of mice to express CMKLR1 has no impact on the development of lung injury, lung inflammation, or AHR induced by acute inhalation exposure to the non‐atopic asthma stimulus, O_3_. However, we report that either CMKLR1 or its ligands (RvE1 and chemerin) significantly limit the severity of innate airway responsiveness to methacholine. Given the heterogenous nature of the asthma phenotype and given that AHR is a cardinal feature of asthma, it is important to pursue further studies to determine if CMKLR1 or its ligands impact the outcome of other asthma phenotypes such as those elicited by antigen sensitization and challenge or obesity.

## FUNDING INFORMATION

The research reported in this publication was supported, in part, by the National Institute of Environmental Health Sciences of the National Institutes of Health under award numbers R03ES022378 (to R.A. Johnston) and R03ES024883 (to R.A. Johnston), the National Institute of Allergy and Infectious Diseases of the National Institutes of Health under award number R03AI107432 (to R.A. Johnston).

## DISCLOSURES

The findings and conclusions in this report are those of the authors and do not necessarily represent the official position of the National Institute for Occupational Safety and Health, Centers for Disease Control and Prevention. Furthermore, the content contained within this publication is solely the responsibility of the authors and does not necessarily represent the official views of the National Institutes of Health.

## ETHICS STATEMENT

The care and use of all animals adhered to the guidelines set forth by the National Institutes of Health (Bethesda, MD) while each of the experimental protocols used in this study was approved by the Animal Welfare Committee of The University of Texas Health Science Center at Houston (Houston, TX, USA; Protocol Number: 15‐0110‐AWC).

## Data Availability

Raw data used to calculate means and standard deviations presented in Tables 1 and 2, Figures 1, 2, 3, 4, 5, and the text are publicly available on the NIOSH Data and Statistics Gateway under Dataset Number RD‐1075‐2023‐0.
